# Photonic crystal fiber sensors in the THz domain: A leap forward in alcohol detection

**DOI:** 10.1016/j.heliyon.2024.e40945

**Published:** 2024-12-06

**Authors:** Most. Momtahina Bani, Khalid Sifulla Noor, A.H.M. Iftekharul Ferdous, Md. Asaduzzaman Shobug

**Affiliations:** Department of Electrical and Electronic Engineering, Pabna University of Science and Technology, Pabna, 6600, Pabna, Bangladesh

**Keywords:** Photonic crystal fiber (PCF), Effective area (EA), Dispersion (DP), Refractive index (RI), Alcohol, Frequency (FR)

## Abstract

Methanol (CH₃OH) is a volatile, transparent, and toxic substance widely used in chemical substrates, antifreeze, and industrial applications. Ethanol (C₂H₅OH), in contrast, is commonly used in alcoholic beverages, as a fuel additive, and as an antiseptic. Differentiating between methanol and ethanol is critical due to the severe health risks associated with methanol ingestion, while ethanol is safe for consumption in moderation. To tackle this challenge, we present a highly sensitive and accurate Photonic Crystal Fiber (PCF) sensor designed for the detection of both methanol and ethanol. The proposed sensor demonstrates impressive performance, with maximum relative sensitivities (RS) of 95.72 % for methanol and 97.55 % for ethanol, while operating within the 2.2 to 3.2 THz range. Additionally, it achieves low Effective Material Loss (EML) values of 0.0066 cm⁻^1^ for ethanol and 0.0044 cm⁻^1^ for methanol, with Numerical Aperture (NA) values of 0.257 and 0.270, respectively. The key advantages of this sensor include its exceptional precision, high sensitivity, and low material loss, making it a reliable solution for accurately distinguishing between methanol and ethanol in various industrial and commercial applications. By providing enhanced detection capability in the THz range, this sensor improves safety monitoring and quality control processes in sectors where these substances are frequently used.

## Highlights

• Differentiating methanol and ethanol is crucial due to methanol's severe toxicity.
• The PCF sensor achieves 95.72 % and 97.55 % RS for methanol and ethanol, respectively.
• Low EML values: 0.0066 cm⁻^1^ for ethanol, 0.0044 cm⁻^1^ for methanol in 2.2–3.2 THz.
• Sensor enhances detection, improving safety monitoring and quality control processes.

## 1 Introduction

A novel optical fiber with micro-structured architecture, PCF, shows a longitudinal periodic pattern of air holes. Due to its unique architecture, the PCF exactly manages its optical properties, such as photon incarceration, variation, and nonlinear behavior. Because of its unique laser-guiding qualities, with features of configurable architecture, the PCF provides advantages such as superior light instruction, customizable dispersion features, and the possibility of a range of diversified uses like detecting telecommunications, and nonlinear optical technologies [1,2]. There are very many applications of PCF across various disciplines (M. [3]) (S.K. [4]). The communication sector benefits from PCF by allowing faster data transmission while reducing signal distortion and dispersion. For sensing applications, PCF can be very useful because of its excellent reactivity to changes in the external environment, like temperature, pressure, and chemical concentrations. PCF provides accurate light delivery and imaging capabilities in healthcare imaging and laser-induced surgery. PCF finds applications in quantum physics, specifically in photon manipulation for quantum information processing and nonlinear optics for the generation of ultrafast pulses (J. Broeng et al., 1991) (J. [5]).

THz radiation, in PCF, deals with the guiding and handling of electromagnetic waves in the frequency range of 0.1–10 THz. THz transmission is highly restricted along with low loss due to micro-structured air pores in PCFs (H. [6]; [null]). Due to the given property, the potential of THz spectral analysis, scans, and communication-related fields is huge with PCFs (H. [7]) (F.M. [8]). PCFs can be designed to produce effective and compact THz generators and sensors, due to which the potential of these new approaches in noninvasive material testing, healthcare diagnosis, and safety inspection has become very high (A.Y. [9]) (L. [10]) (J. [11]). A gas analyzer uses the special qualities of PCF to measure the amount of gas present by examining how illumination interacts with its molecules inside the fiber. Zhang Zhi-guo et al. proposed, "Gas sensing characteristics of the index-directed PCF using an air core" (Z. [12]). Simon alongside other fellows demonstrated, "Ideal PCF narrows for super continuum emitters boosted by blue" [13]. To precisely monitor temperature swings, a PCF temperature gauge makes use of the fundamental properties of the PCF or heat-dependent fluctuations in its RI. Guolu as well as his mate innovated, "Refractive index and temperature measurements simultaneously using liquid-filled PCF and LPFG" (G. [14]). A pair parallel cores under each PCF framework make up a dual-core PCF detector, which enables asymmetric assessment for improved perception. Shanyong et al. proposed, "Transformation of Spatial Modes using Hybrid Dual-Core Photonic Crystal Fiber" (S. [15]).

A PCF velocity tracker uses the relation amongst radiation and the turbulent media inside the PCF to estimate the motion of liquids or things that are moving. Khurram among his fellows suggested, "Two-dimensional bending Velocity Detector with Excellent Sensitivity Using an Elliptic Two-Core PCF" (K. [16]). Once placed under illumination, an object's surface, usually made of Au or Ag, and may vary its RI. This can be measured using surface plasmon resonance (SPR), a type of monitoring approach. SPR are produced whenever polarized radiation strikes the metallic layer at an exact point and causes its free ions to vibrate. Jian-Ying et al. performed, "Refractive index detecting properties of a photonic crystal fiber SPR sensor coated with carbon nanotubes" (J.Y. [17]). Two distinct radiation waves are used by a dual-wavelength enabled PCF device to improve sensitivity. It may assess several variables either linearly or in tandem by using numerous wavelengths, which increases the instrument's flexibility along with applicability. Inaki plus his fellows invented, "A Modular Dual-Wavelength Locked Fiber Laser Source with Switch ability for In-PCF Parametric Frequency Modulation in CARS Microscopy" (I. [18]). A hollow-core PCF (HC-PCF) monitor makes use of a special construction in which the centre of a fiber is encircled by a regular pattern of air gaps. The radiation can go across the HC in this layout. Nikoleta alongside fellows found, "Rapid Molecule Spectroscopy Employing a Hollow-Core Photonic Crystal Fiber Light" (N. [19]). A PCF biosensor detects biomolecular contacts by taking advantage of the special qualities of PCF. They enable exceptional responsiveness, label-free recognition, including immediate tracking by analyzing shifts in optical conveyance or resonant throughout the fibre induced by adsorption interactions. As such, they are essential resources in bioengineering along with healthcare. Iddrisu et al. preferred, "PCF-based SPR refractive index biosensor assaying: from new setups to exceptional detection limits" (I. [20]). The exact regulate of how photon travels is made possible by a hexagonal structure that contains air spaces encircling the center of a hexagonal PCF device. The accuracy and versatility of the scanner to determine different chemical alongside physical variables are improved by this construction. Shuvo et al. innovated, "An optical sensor based on hexagonal photonic crystal fibers (H-PCF) with minimal confinement loss and high relative sensitivity for the terahertz (THz) regime" (S. [21]). Mohammad as well as his partner suggested, "High-sensitivity gold-coated PCF biosensor with surface plasmon resonance technology" (M.R. [22]). The RI is a key factor in PCF since it controls and directs photons. The radiation may be contained alongside steered inside the interior of a fibre thanks to the sequential configuration of air spaces that either produces a bandgap of photons or alters the effective RI. Xili Jing alongside his mates performed," A PCF coated in silver that serves as the basis for a broadband SPR dual-channel sensor for temperature and refractive index measurements" (Z. [23]). Petrochemicals are crucially processed goods that utilize oil from petroleum, including gasoline, petrol as well as diesel. In several areas of the earth, gasoline is a necessity for both home and commercial use since it is often utilized as aviation fuel along with warmth and illumination purposes in lanterns alongside burners. Petroleum, often known as petrol, is an essential component of the automotive sector since it is largely utilized as oil for the engines with combustion in automobiles. In addition to powering a variety of vehicles, such as automobiles, buses, and trucks, diesel is a necessary component of commercial along with farming equipment. Diesel-powered vehicles run on this fuel. A. H. M. Iftekharul with his fellows demonstrated, "Using terahertz spectrum analysis to reveal new information about oil derivatives: the hybrid refractive index rectangle core photonic crystal fiber viewpoint sensing" [24]. Khalid et al. offered, "Terahertz spectrum petrochemical sensing: a hybrid structure photonic crystal fiber refractive index method" (K.S. [25]). Md. Asaduzzamzn among fellows proposed, "Fuel quality assurance using terahertz region operation management and hybrid hexagonal circular hollow core PCF sensing" (M.A. [26]). R. Kanmani et al. suggested, "The terahertz spectrum effectiveness of providing core components for slotted core Q in PCF" (R. [27]). The distinctive characteristics of the fiber composition are used by a PCF chemical analyzer to identify and evaluate chemical elements. Such gadget provide excellent responsiveness, effectiveness, along with immediate tracking skills by taking advantage of variations in the movement of light or resonant inside the fiber brought about by connections to particular molecules. They enable quick and precise chemical ingredient analysis in a variety of domains, including process manufacturing oversight, clinical tests, along with surveillance of the environment. Kawsar with his partner established, "Creation of an optical sensor utilizing terahertz spectroscopy for chemical detection" (A. Kawsar et al., 2019). Diponkar Kundu et al. innovated, "Analyzing the Terahertz photonic crystal fiber sensor's performance in chemical detection" (D. [28]).

Methanol is sometimes referred to as wood alcohol or methyl alcohol. It smells slightly pleasant and is a colorless, uncertain, burning solvent. It is extremely harmful to living things and may trigger serious illnesses, like as cataracts, organ harm, or even death, if consumed, inhaled, or absorbed through skin (J. Ott et al., 2024). Ethanol, sometimes referred to as alcohol or ethyl alcohol. It is a dynamic, ignited, transparent solution having a distinct flavour and smell that burns [29]. Ethanol is harmless for humans to consume in moderation, compared to methanol. Because methanol and ethanol have rather distinct impacts on people's health and are used for very distinct purposes, it is important to distinguish between the two because methanol is extremely harmful that may trigger major health problems [30]. In recent years, various techniques have been explored for methanol detection, including surface plasmon resonance (SPR) sensors, fiber optic sensors, and electrochemical methods. While these approaches offer some advantages, they often suffer from limitations such as lower sensitivity, reduced accuracy, or limited operational range. Photonic Crystal Fiber (PCF) sensors have gained significant attention due to their high sensitivity, low material loss, and ability to operate in the THz range, making them a promising solution for methanol detection in industrial and commercial applications where precision and safety are crucial. The structural properties of PCF sensors, such as their unique core design and air-hole arrangement, significantly enhance performance in the THz domain for alcohol detection (Z. [31]). These features improve light confinement and reduce loss, allowing efficient propagation of THz waves through the fiber. The high porosity of the PCF structure increases interaction between the guided light and the analytes (methanol and ethanol), boosting sensitivity to refractive index changes. This structural optimization is critical for achieving high detection accuracy and low material loss in the THz range. THz PCF sensors offer several advantages over traditional alcohol detection methods. They provide higher sensitivity and selectivity by leveraging the unique absorption characteristics of alcohols in the THz range, enabling precise identification of methanol and ethanol. The photonic crystal structure enhances light-matter interaction, improving detection accuracy. Additionally, PCF sensors operate non-invasively and require minimal sample preparation, making them faster and more efficient than conventional chemical or chromatographic methods. Their compact design also allows for real-time monitoring in various industrial and safety applications.

**Fig. 1:**
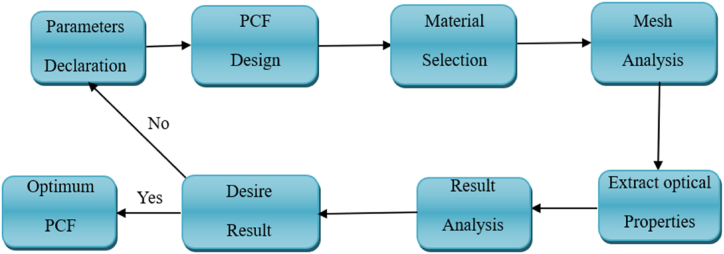
Schematic of experimental setup for proposed PCF sensor.

To address the critical need for distinguishing between methanol and ethanol, we propose a Photonic Crystal Fiber (PCF) sensor designed to operate in the THz range. This sensor leverages the unique properties of PCF, such as enhanced light confinement and low-loss propagation, to achieve high sensitivity in detecting refractive index variations associated with different alcohols. With its tailored heptagonal core structure, the sensor is optimized for real-time alcohol detection, making it suitable for both industrial and commercial applications where precise chemical identification is essential. This design not only improves detection accuracy but also enhances safety monitoring, offering a robust solution for environments where methanol contamination or misidentification can pose significant health risks. Fig. 1 illustrates the block diagram of the setup used to test the sensor's performance in detecting alcohol. This figure provides a schematic of the PCF sensor experimental setup, essential for detecting methanol and ethanol in controlled conditions. The setup enables a systematic investigation into how the sensor performs under varying alcohol concentrations and frequencies.

## 2 Methodology

**Fig. 2:**
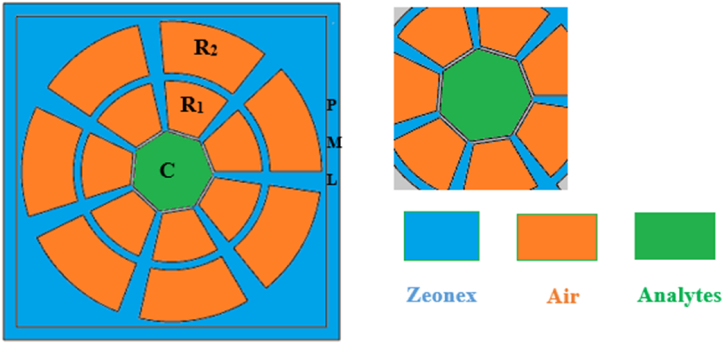

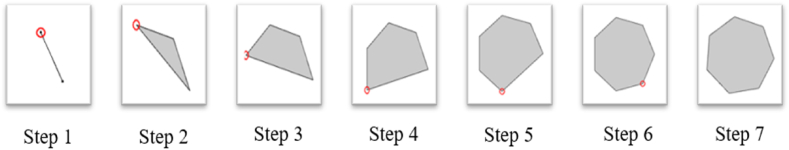

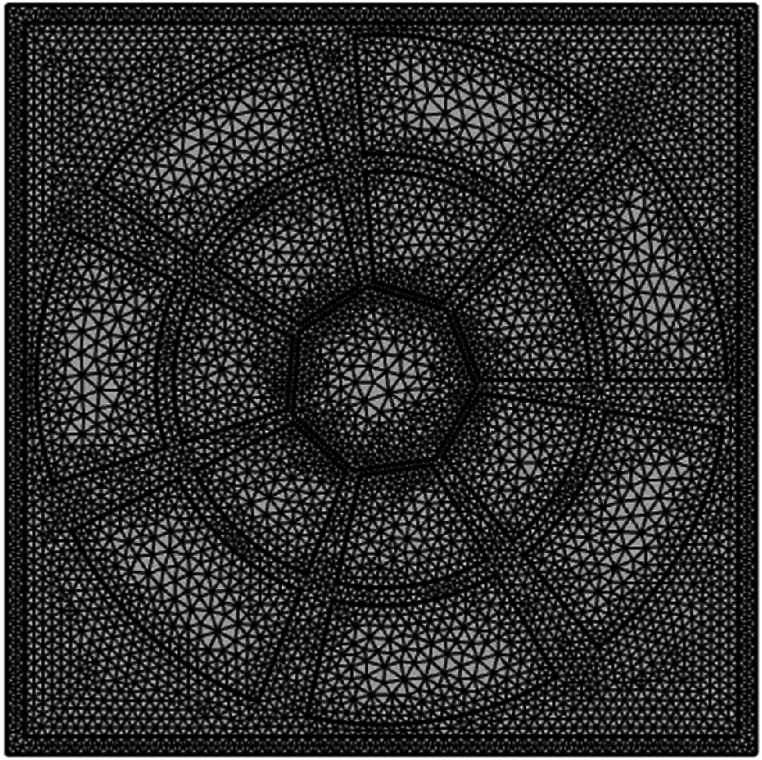

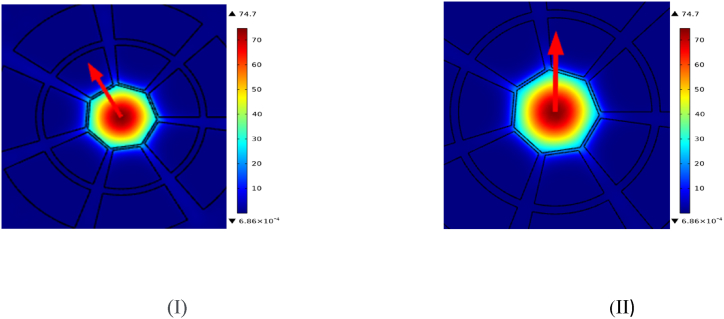

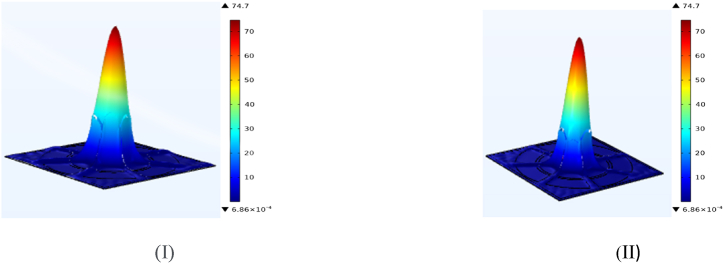

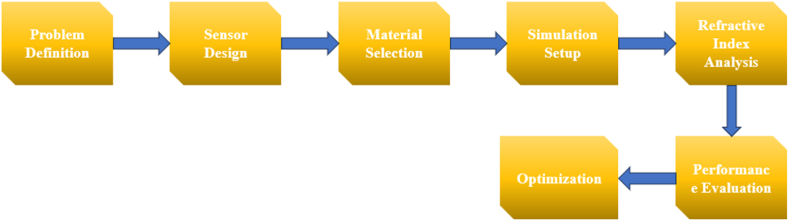
a): Cross-sectional view of the proposed heptagonal core PCF sensor. Fig. 2(b): Step-by-step construction of proposed heptagonal core. Fig. 2(c): Mesh representation of the proposed PCF sensor. Fig. 2(d): Power density distribution for (I) methanol (II) ethanol Fig. 2(e): Power confinement analysis in core and cladding for (I) methanol (II) ethanol Fig. 2(f): Workflow for material selection, sample preparation, and analysis.

The first process of developing the PCF sensor using COMSOL Multiphysics involves determining as well as characterizing the size along with components of the PCF layout. Recent advancements in photonic crystal technology and THz source development have significantly improved the efficiency of PCF sensors by enhancing light confinement and reducing losses. Innovations in THz generation and detection have also lowered costs, making the sensors more affordable for practical applications. These developments have broadened the scope of PCF sensors for various industries, including alcohol detection, with better performance and accessibility. This model tries to simulate the energy transmission procedure through chemical action comprising alcohol (Methanol, Ethanol) and the exterior operation of the PCF using COMSOL Multiphysics for alcohol identification through PCF. We construct this new device and examine it with COMSOL Multiphysics. This PCF has an unusual heptagonal core, a unique heptagonal clad surface, with 14 airways (R_1_ & R_2_) that originate within the cladding. The heptagonal core is designed considering polygonal function of COMSOL Multiphysics alongside desired angle of 51°. Whereas 14 airways are created in two cycles. R_2_ be the radius of outer circle that we have use to create the outer layer airholes by using difference function. Similarly, the inner airways cycle consists of 7 similar airways that are created in similar manner but instead of circle we consider heptagon with R_1_ radius for this portion and also use difference function here to achieve this shape. Zeonex is the base material used for the suggested system. Suggested THz PCF sensors can distinguish between different types of alcohols by detecting their unique refractive index and absorption properties in the THz range. This capability enhances practical applications, such as preventing methanol contamination in industrial settings and ensuring public safety by accurately identifying hazardous alcohols in real time. Zeonex is a material with a pre-calculated Refractive Index (RI) of 1.53. But its core (C) uses RI to detect Alcohol, where RI for methanol and ethanol are 1.329,1.361 respectively. A Perfectly Matched Layer, or PML for short, is a computing borders that simulates proper instrument behaviour by absorbing outbound pulses as well as preventing rebounds. Here, Fig. 2(a) Displays the cross-sectional structure, showing the heptagonal core and air holes that facilitate alcohol detection. The cross-sectional view of the heptagonal core structure reveals how air holes are arranged around the core, which enhances light confinement. This layout is crucial for achieving high sensitivity in detecting refractive index differences between methanol and ethanol. Fig. 2(b) Depicts the stages in designing the heptagonal core structure of the PCF sensor. This figure illustrates the construction process of the heptagonal core, showing the layered assembly of air holes that optimize sensitivity. Each step in the construction enhances the sensor's ability to differentiate between analytes based on their refractive indices.

The discretized description of detector topology used in calculations is known as a mesh representation. Such a mesh consists of minor elements like triangular and quadrilaterals, which when combined roughly, may represent the intricate framework inside the PCF. The use of triangular and quadrilateral elements in meshing is vital for accurate simulation of our heptagonal core PCF sensor. Triangular elements are applied to irregular regions, while quadrilateral elements cover more structured areas, ensuring an optimal balance between precision and computational efficiency. This hybrid meshing improves convergence and reduces numerical errors, enhancing the accuracy of key parameters like Relative Sensitivity (RS) and Effective Material Loss (EML). As a result, the mesh ensures reliable analysis and performance of the sensor for methanol and ethanol detection. The principles regarding the transmission of light can be solved by techniques like the FEM, working by breaking the detector down to such functional components. There are 74 apex factors, 1309 border factors, and 11228 items in total. The minimal grade for a substance is 0.5861. Upon which Fig. 2(c) Shows the meshing layout used in the simulation, illustrating triangular and quadrilateral elements for accuracy. The meshing diagram used in simulations shows how triangular and quadrilateral elements cover the PCF structure. This meshing improves computational accuracy, particularly in simulating the interaction between light and analytes within the core.

A PCF sensor's power distribution explains how the power of light is dispersed evenly throughout a fibre's topology. Because of its cladding with sequential patterns of air pores, radiation within a PCF is firmly constrained inside its core and improves guidance via adjusted TIR, or optoelectronic bands. Extreme reactivity to fluctuations in the outside world is created via an evanescent area that extends through the panelling and primarily operates in a basic pattern. The spacing is often analyzed through computer modeling and testing, so as to optimize layout as well as functioning. It is of prime importance to the effectiveness of the sensor. Upon which Fig. 2(d) depicts Visual representation of the power density distribution across the sensor when detecting methanol (I) and ethanol (II). showing how the design effectively confines light, which is critical for enhancing sensitivity to each analyte.

The PCF sensor density distribution is explained by the changes of RI in the core and the surrounding. Energy is mostly confined by the higher RI of the core rather than the cladding. This configuration affects the guiding of illumination, modal area confinement, and the susceptibility to external perturbations of importance to the optimization of detector performance for a wide range of applications. It is often periodic due to the designs of air vents in the flow. Upon which Fig. 2(e) Shows power confinement in the core and surrounding cladding for methanol (I) and ethanol (II), demonstrating the sensor's sensitivity. emphasizing the sensor's capability to guide light with minimal leakage, a feature essential for precise detection.

The simulation results indicated differences in Effective Area (EA) and spot sizes (SP), with EA values of 3.92 × 10⁻⁸ m^2^ for ethanol and 3.54 × 10⁻⁸ m^2^ for methanol, and SP values of 2.28 × 10⁻⁴ μm for ethanol and 2.12 × 10⁻⁴ μm for methanol. While these numerical differences suggest distinct optical properties for each analyte, a formal significance analysis was not performed since the data is derived from deterministic simulations rather than experimental iterations. In future work, we plan to incorporate sensitivity and uncertainty analyses to evaluate the significance of these differences. This will help us better understand how small parameter variations could influence the sensor's performance and ensure reliable detection of methanol and ethanol in real-world applications.

The design process for a heptagonal core Photonic Crystal Fiber (PCF) sensor begins with defining the problem, specifically the need for detecting methanol and ethanol based on their refractive indices to ensure safety and monitoring in various industries. The sensor is then designed with a focus on the heptagonal core structure, shape, and arrangement of air holes. Appropriate materials for the core and cladding, such as Zeonex or silica, are selected based on their refractive indices to ensure compatibility with THz sensing. The next step involves setting up the sensor simulation in COMSOL Multiphysics, configuring parameters like pitch, air hole diameter, and the operational THz range. The refractive indices of methanol and ethanol are inputted into the simulation to analyze their effects on light propagation through the sensor. The sensor's performance is evaluated based on key metrics, including Relative Sensitivity, Effective Material Loss, Confinement Loss, and Numerical Aperture. Finally, the sensor design is optimized by tweaking the geometrical parameters to achieve maximum sensitivity and low loss for both methanol and ethanol detection. Fig. 2 (f) Outlines the procedural flow from material selection to sample analysis for the PCF sensor. Outlines the comprehensive process from material selection to sample analysis, ensuring that each phase is optimized for high-performance detection in commercial and industrial settings.

## 3 Results and discussion

### 3.1 Numerical analysis

Finite Element Method (FEM) is a computational method applied in science and technology for the solution of awkward problems. Simulation of optical characteristics and light transmission in PCFs utilizes FEM. Special visual features of PCF include adjustable divergence and strong ambiguity due to the sequential placement of ventilation pores throughout their length. FEM enables the segmentation of longitudinal shapes into PCF pieces, where the characteristics can be analyzed with accuracy; this way, it becomes easy to research electrical field dispersion, together with the general effectiveness of the fiber.

Relative Sensitivity (RS) is a property that gauges the capacity of PCFs to limit alongside direct light in response to variations in the RI of their surrounding environment. On PCFs, there is a repeating pattern of air vents that influences the characteristics of guiding. Photon containment and the fiber's acoustic characteristics are measured by RS, which also measures changes in these holes or the RI difference. High RS in PCFs is essential for purposes such as recognition and telecommunications since it assures proper light direction alongside the responsiveness of environmental changes, giving the fiber fitter the ability to identify and quantify the impacts in the surroundings.

Implement those computational equations to determine RS [24].

(1) $$r=\frac{n_{r}}{n_{eff}}\times p\%$$

Nevertheless, P occupies its rightful place in identifying a member of RI.

(2) $$p=\frac{\int_{sample}R_{e}\left(E_{x}H_{y}-E_{y}H_{x}\right)dxdy}{\int_{total}R_{e}\left(E_{x}H_{y}-E_{y}H_{x}\right)dxdy}$$

Conversely, quantities in MF are delimited by *H*_*x*_, while quantities in EF are conveyed by *E*_*x*_ along a surface.

**Fig. 3:**
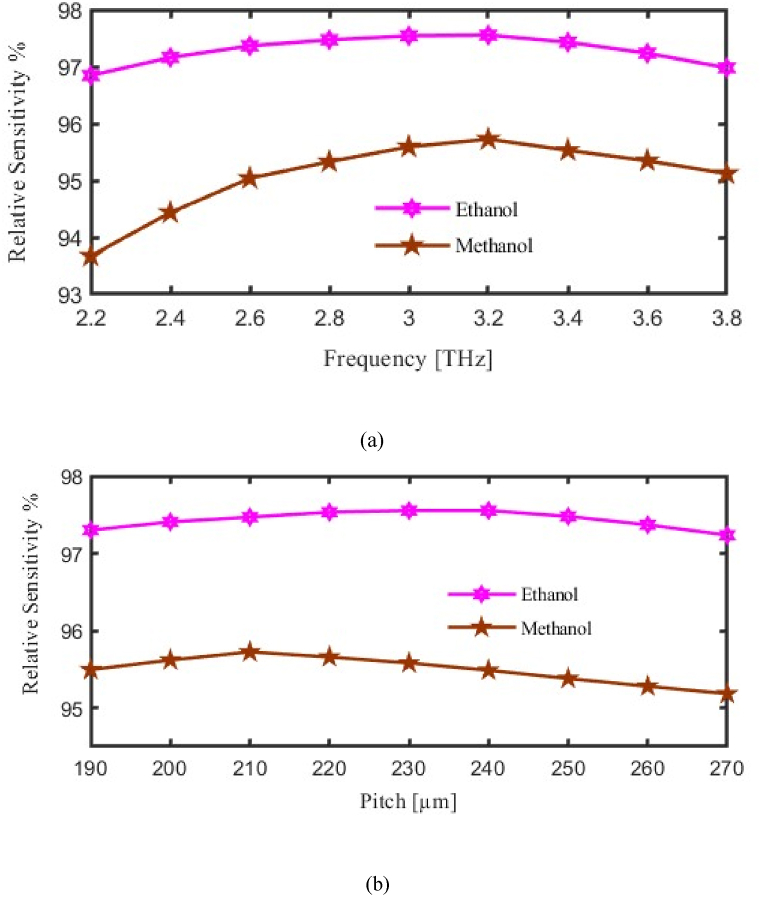
Demonstrate the influence of the Relative Sensitivity (a) Frequency [THz] (b) Pitch [μm].

Firstly, we measured the RS about both P and FR at the same time. The vertical axis to be considered for RS and horizontal axis considered for FR or P. As we made variation in FR or in P then corresponding change will occur in the RS. Here pink curve for ethanol and brown curve for methanol represent the RS. Fig. 3(a) Shows the relationship between frequency and relative sensitivity, demonstrating that the sensor achieves peak sensitivity at 3.2 THz, which is key for reliable detection of methanol and ethanol. Where the max RS for methanol and ethanol is 95.72 % and 97.55 % at f = 3.2 THz. By contrast, Fig. 3(b) Examines how pitch affects sensitivity, revealing that certain pitch values enhance the sensor's ability to detect subtle differences in refractive indices, optimizing performance for each analyte.

In PCF, Effective Material Loss (EML) describes light retardation resulting from the fiber element's natural acceptance, silica, for example. The EML in PCFs depends on light restriction in the core and its interaction with the surrounding micro-structured cladding. Light interaction intensity with the fiber material depends on several things, which include the wavelength used and the air-hole layout concept. EML is one of the most important factors when it comes to determining the total loss for PCFs. It also affects how suitable and efficient they are for various applications, from sensors to telecommunications.

Implement those computational equations to determine EML [24].

(3) $$\alpha_{eff}=\frac{{\left(\frac{\varepsilon_{0}}{\mu_{0}}\right)}^{\frac{1}{2}}\;\int_{A_{max}}n\alpha_{mat}{\left|E\right|}^{2}dA}{2\int_{ALL}S_{z}dA}$$

Whereas the electric field is represented by E and the zeonex loss coefficient is $\alpha_{mat}$.

**Fig. 4:**
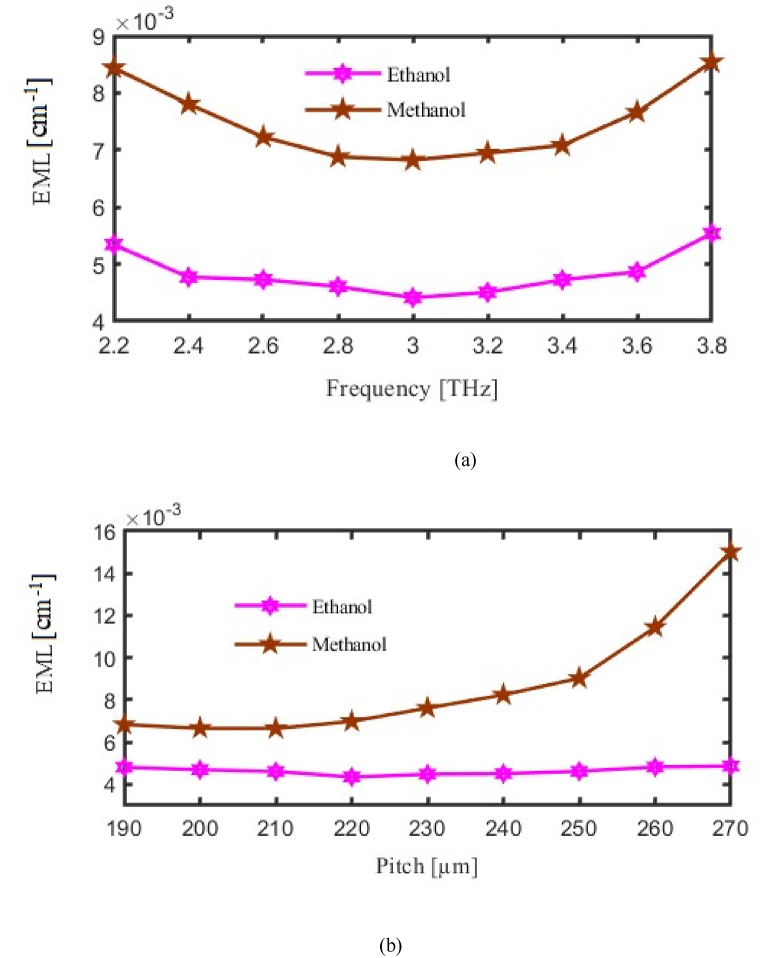
Demonstrate the influence of the Effective Material Loss (a) Frequency [THz] (b) Pitch [μm].

We are now exploring the EML of our proposed PCF. The relationship between EML, pitch, and operational frequency is highlighted in Fig. 4. The vertical axis to be considered for EML and horizontal axis considered for FR or P. As we made variation in FR or in P then corresponding change will occur in the EML. Here pink curve for ethanol and brown curve for methanol represent EML. Where at f = 3.2 THz the EML finds 0.0066 cm⁻^1^ and 0.0044 cm⁻^1^ for methanol and ethanol respectively. To be more specific , Fig. 4(a) Analyzes Effective Material Loss (EML) across frequencies, demonstrating the sensor's efficiency in retaining light within the core for both methanol and ethanol, which directly influences detection accuracy, while Fig. 4(b) Illustrates the influence of pitch on EML, showing that an optimal pitch configuration minimizes loss, thereby enhancing light confinement and the sensor's overall sensitivity.

An indicator of a PCF's capability of keeping photons within its core is Numerical Aperture (NA). It measures the spectrum of orientations at which the fiber can support photons and is dependent on the RI difference between the cladding and core. The microstructure configuration of air holes surrounding the core of PCFs determines the NA, and these holes can be tailored for delivering certain light-guiding characteristics. An increased NA, for systems that require precise modal containment and efficient transmission of light, indicates a stronger ability to capture and steer photons.

Implement those computational equations to determine NA [24].

(5) $$NA=\frac{1}{\sqrt{1+\frac{{\pi A}_{eff}f^{2}}{c^{2}}}}\approx \frac{1}{\sqrt{1+\frac{{\pi A}_{eff}}{\lambda^{2}}}}$$

Utility wavelengths are represented by λ, while the PCF's EA is displayed by A_eff_.

**Fig. 5:**
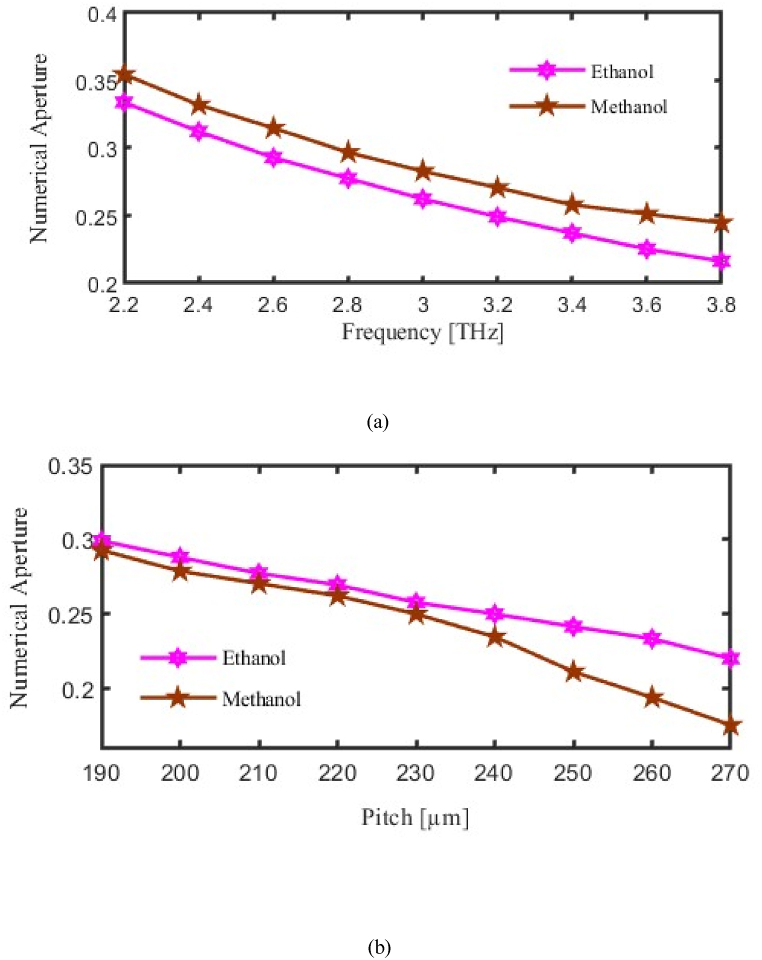
Demonstrate the influence of the Numerical Aperture (a) Frequency [THz] (b) Pitch [μm].

As seen in Fig. 5, the NA of the suggested PCF is examined by varying the pitch and frequency. The vertical axis to be considered for NA and horizontal axis considered for FR or P. As we made variation in FR or in P then corresponding change will occur in the NA. Here pink curve for ethanol and brown curve for methanol represent NA. Where at f = 3.2 THz the NA finds 0.270 and 0.257 for methanol and ethanol respectively. Fig. 5(a) Shows How Numerical Aperture (NA) varies with frequency, indicating that the sensor captures light most effectively at specific THz frequencies, which is important for maximizing detection reliability. In the meantime, Fig. 5(b) Examines the effect of pitch on NA, highlighting that a suitable pitch value enhances the sensor's photon capture, improving its responsiveness to changes in refractive index.

The Effective Area (EA) of PCFs is a measure of the cross-sectional region over which light is efficiently directed and contained in the fiber core. The EA is a parameter that impacts some of the important characteristics, such as dispersion and nonlinearity. The higher light intensity inside the core due to a small EA would thus enhance nonlinear effects and hence be useful for applications like supercontinuum generation. At the same time, a larger EA is preferred when high-power transmission is required since it reduces nonlinearity. The EA is defined by the core size and the configuration of air holes in the cladding, which can be modified to tailor the optical characteristics of the fiber for specific applications.

Implement those computational equations to determine EA [24].

(6) $$A_{eff}=\frac{{\left[\int I\left(r\right)rdr\right]}^{2}}{{\left[\int I^{2}\left(r\right)rdr\right]}^{2}}$$

Consequently, the E.field is displayed over the identifying component when I(r) = |E|^2^.

**Fig. 6:**
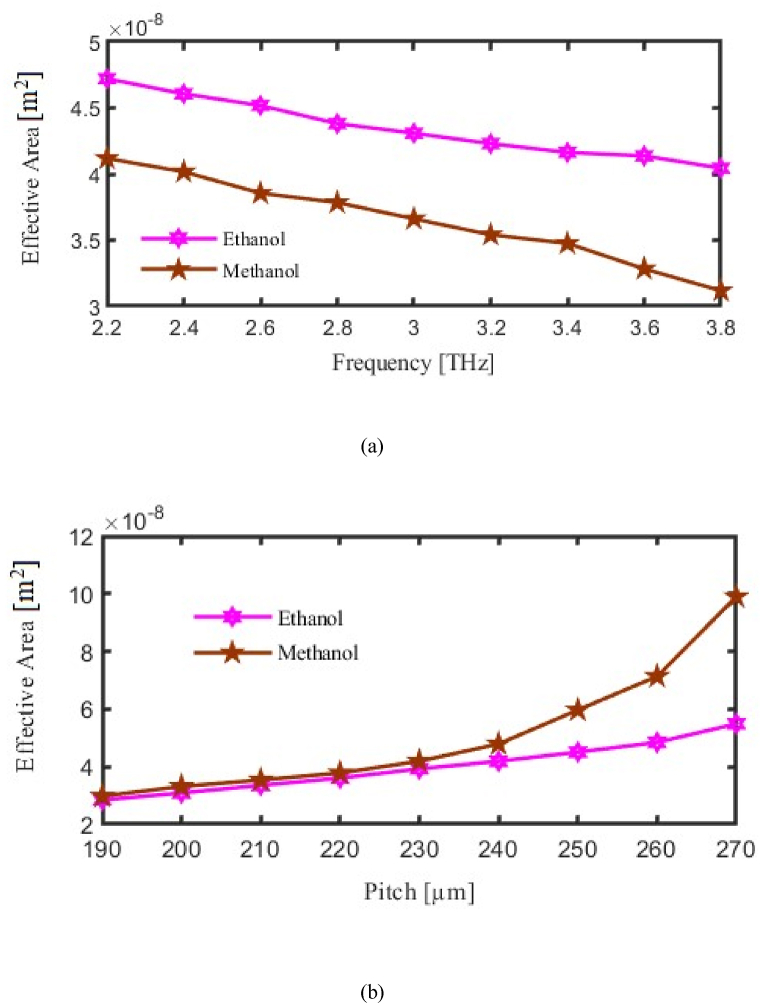
Demonstrate the influence of the Effective Area (a) Frequency [THz] (b) Pitch [μm].

The present experiment investigates the link between EA and changes in the PCF's operating frequency and pitch. The vertical axis to be considered for EA and horizontal axis considered for FR or P. As we made variation in FR or in P then corresponding change will occur in the EA. Here pink curve for ethanol and brown curve for methanol represent EA. Where at f = 3.2 THz the EA finds 3.54 × 10⁻⁸ m^2^ and 3.92 × 10⁻⁸ m^2^ for methanol and ethanol respectively. The dependence of EA on frequency is shown in Fig. 6(a), Investigates the effective area as a function of frequency, illustrating that controlling the core area is essential for achieving optimal light confinement and detection sensitivity. In addition, Fig. 6(b) Shows the effect of pitch variation on the effective area, revealing that pitch adjustments help optimize the core's ability to contain light and increase the sensor's sensitivity to analytes.

In PCF, the Spot Size (SP) is the effective diameter of the optical mode enclosed in the fiber core. It is an important factor that influences the efficiency of coupling, bending losses, and modal dispersion of the light transmission. The core size and the configuration of the air pores in the cover define the spot size. In asymmetric photonic programs, smaller spot sizes lead to higher intensity and better phase confinement. However, in applications that need significant power, larger spot sizes minimize discontinuity, thereby improving the excellence of the beam and, consequently, decreasing the intensity. Using the PCF layout can modify the spot size to meet certain requirements regarding performance.

Implement those computational equations to determine Spot size [24].

(7) $$W_{eff}=R\times \left(.65\times {1.619\times V}^{-1.5}+{2.789\times V}^{-6}\right)$$

Whenever the altered FR band V and heptagonal core diameter R are used together.

**Fig. 7:**
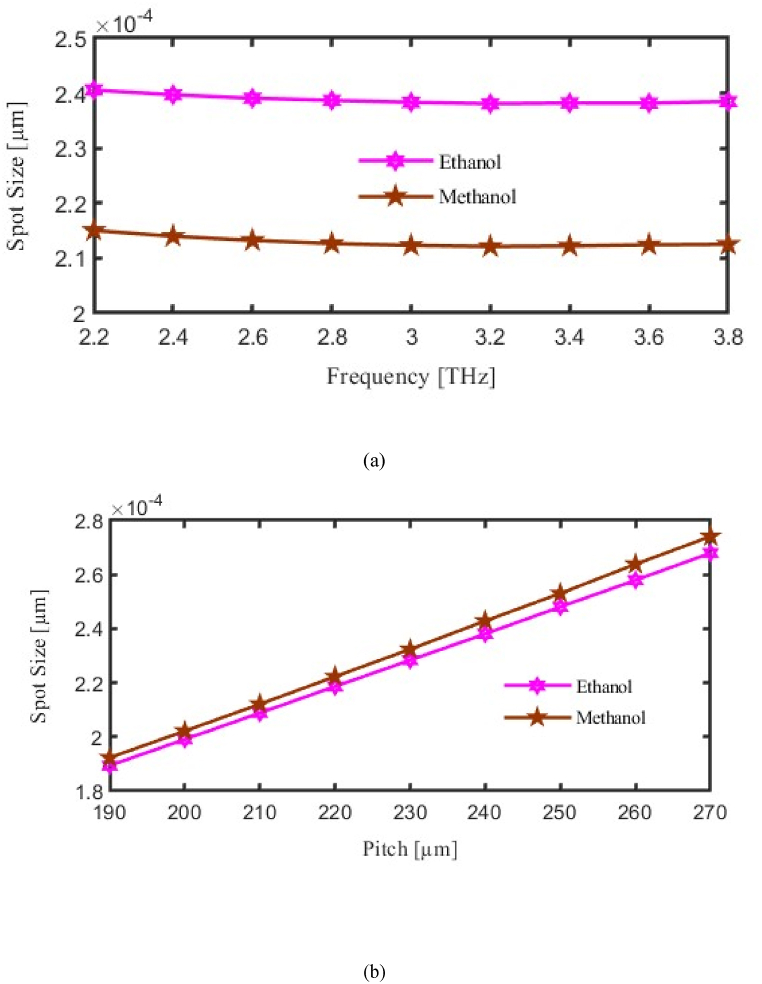
Demonstrate the influence of the Spot size (a) Frequency [THz] (b) Pitch [μm].

Illustrated As in Fig. 7, this section explores the relationship between pitch, operating frequencies, FR, and spot size. The vertical axis to be considered for SP and horizontal axis considered for FR or P. As we made variation in FR or in P then corresponding change will occur in the SP. Here pink curve for ethanol and brown curve for methanol represent SP. Where at f = 3.2 THz the SP finds 2.12 × 10⁻⁴ μm and 2.28 × 10⁻⁴ μm for methanol and ethanol respectively. Fig. 7(a) Illustrates the variation of spot size with frequency, demonstrating that smaller spot sizes improve light confinement within the core, critical for achieving high detection accuracy. Conversely, Fig. 7(b) Examines the effect of pitch on spot size, showing that fine-tuning the pitch results in an ideal spot size that maintains the balance between confinement and light intensity.

The term Dispersion (DP) of light in a PCF instrument indicates that the unique geometry of the fiber causes its velocity of a beam to change with its wavelengths. PCFs may exhibit abnormal or normal dispersion, depending on the specifications of their structure. Briefer wavelengths transmit more quickly under ordinary variation, but abnormal dispersion has the reverse impact.

Implement those computational equations to determine Dispersion of light [24].

(8) $$\beta_{2}=\frac{2}{c}\;\frac{d}{d\omega }\;\left(n_{eff}\right)+\frac{\omega }{c}\;\frac{d^{2}}{{d\omega }^{2}}\;\left(n_{eff}\right)$$

Here, $\beta_{2}$ represents dispersion parameter, *c* be the velocity of light, ω be the angular frequency.

**Fig. 8:**
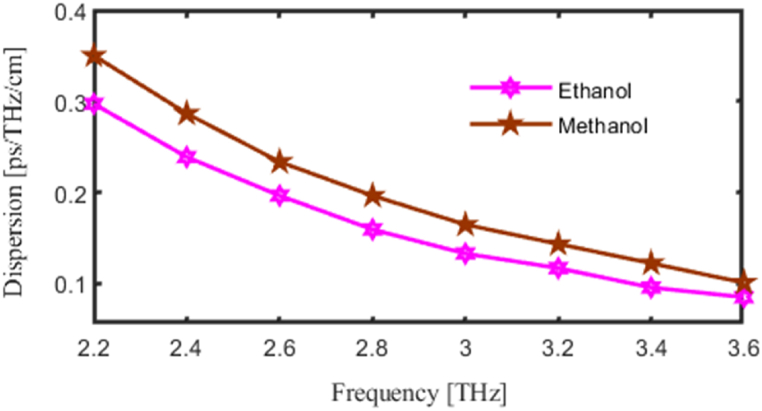
Demonstrate the influence of the Dispersion of light due to Frequency [THz].

This section explore relation between light dispersion with the operating frequency of this suggested PCF sensor. The vertical axis to be considered for DP and horizontal axis considered for FR or P. As we made variation in FR or in P then corresponding change will occur in the DP. Here pink curve for ethanol and brown curve for methanol represents DP. Where at f = 3.2 THz the DP finds 0.2 ps/THz/cm and 0.14 ps/THz/cm for methanol and ethanol respectively. Fig. 8 Provides an analysis of dispersion in the THz range, showing how frequency impacts light speed and, consequently, the sensor's ability to distinguish between different analytes based on dispersion properties.

This section's Table 1 compares the envisioned sensor's functionality to that of several already in consideration. We have mentioned the sensing analytes, operating frequency, RS, EML, NA and EA of those sensors with ours suggested sensor. From the table you can see that the sensing ability or RS of our intended detector is much better than that of others. It also having minimal EML that the mentioned sensors.

**Table 1:** Performance study among currently available detector with our invented detector.

Ref.	Analytes	FR (THz)	RS (%)	EML (cm^−1^)	NA	EA (m^2^)
(R. [27])	Benzene	1.0	74.61	0.0138	–	–
	Ethanol	1.0	74.53	0.0148	–	–
	Water	1.0	74.17	0.0172	–	–
(A. Kawsar et al., 2019)	Water	1.0	73.20	–	–	1.43 × 10^−7^
	Ethanol	1.0	76.44	–	–	1.36 × 10^−7^
	Benzene	1.0	77.16	–	–	1.35 × 10^−7^
(S. [21])	Benzene	1.0	81.46	–	–	–
(M.S. [32])	Ethanol	1.6	85.7	–	0.372	69800 (μm^2^)
(M.A. [33])	Benzene	1.7	89	0.028	0.42	–
(D. [28])	Ethanol	2.0	95.21	0.0078	0.314	6.52 × 10^−8^
	Benzene	2.0	94.67	0.0083	0.307	6.84 × 10^−8^
(M.E. [34])	Water	1.0	96.25	–	–	6.84 × 10^−6^ (μm^2^)
**Present** **Research**	Methanol	3.2	95.72	0.0066	0.270	3.54 × 10^−8^
	Ethanol	3.2	97.55	0.0044	0.257	3.92 × 10^−8^

Our planned chemical sensing to existing PCF monitors is the topic of a structured discussion that we are having. Table 1 provides additional background on our PCF research by detailing the main components of the current PCF type. The proposed methanol detection method using a heptagonal core PCF sensor demonstrates impressive performance when compared to existing works based on key factors such as Relative Sensitivity (RS), Effective Material Loss (EML), Numerical Aperture (NA), and Effective Area (EA). Achieving a maximum RS of 97.55 % for ethanol and 95.72 % for methanol at 3.2 THz, the sensor significantly outperforms other methods in the literature, such as those in (S. [21]) (A. Kawsar et al., 2019) (M.S. [32]), where RS values range between 74.17 % and 85.7 % at lower frequencies (1.0–1.6 THz). These high RS values reflect the sensor's superior ability to detect slight variations in refractive index, which is critical for accurate chemical identification. Moreover, the low EML of 0.0066 cm⁻^1^ and high NA of 0.270 demonstrate superior signal transmission and improved light confinement compared to other techniques that report higher EML or lower NA values, such as in (M.A. [33]) (D. [28]). These advancements not only enhance the sensitivity and optical performance of the proposed sensor but also make it highly suitable for hazardous industrial environments, ensuring accurate methanol detection and reducing the risk of poisoning. Achieving such high sensitivity at 3.2 THz further underscores the sensor's advanced performance in the terahertz range, making it a highly effective solution for industrial monitoring and safety. The previous type is generally composed of a mixture of silicon dioxide and related materials and was particularly developed for constructing PCF measurement devices. Generally, fibers are shaped by the stack-and-draw process, which involves heating the mold before dragging it over a fiber. For specific purposes, perforations can be carved or engraved to assist with scanner engagement. A general function to enhance selectivity and sensitivity is to add functional coatings. Moreover, for individual sensors, detection mechanisms are essential such as microscopic particles present in cell walls (A.I. [35]) (C.G. [36]).

### 3.2 Probable error calculation

Our study is based primarily on simulations conducted using COMSOL Multiphysics, which involve complex numerical calculations that are highly precise. Unlike experimental data, these simulations are deterministic and do not contain the random measurement errors commonly encountered in experimental work. As a result, the use of error bars, typically used to reflect experimental uncertainties, is not as relevant in the context of our simulation-based research. However, we have incorporated numerical equations that allow for the estimation of potential errors for various parameters such as relative sensitivity, confinement loss, effective material loss, numerical aperture, spot size, and effective area. These equations provide a theoretical basis for approximating uncertainties in these parameters. The probable error can be calculated using statistical techniques, particularly during sensor calibration or testing.

The process for calculating probable error involves the following steps.

(1) **Data Collection:** Gather multiple readings from the sensor.
(2) **Mean Calculation:** Compute the average (mean) value by summing all the readings and dividing by the total number of readings (K.S. [25]):

(9) $$\bar{x}=\left(1/n\right)\;\sum_{i=1}^{n}X_{i}$$

(3) **Standard Deviation (SD) Calculation:** Measure the variation of the readings from the mean using the standard deviation formula (K.S. [25]):

(10) $$\sigma =\sqrt{\left(1/n\right)\sum_{i=1}^{n}\left(X_{i}-\bar{x}\right)\exp\;\left(2\right)}$$

(4) **Probable Error (PE) Calculation:** Finally, determine the probable error by multiplying the standard deviation by a constant (0.6745) (K.S. [25]):

(11) PE=0.6745×σ

## 4 Application and limitation

**Fig. 9:**
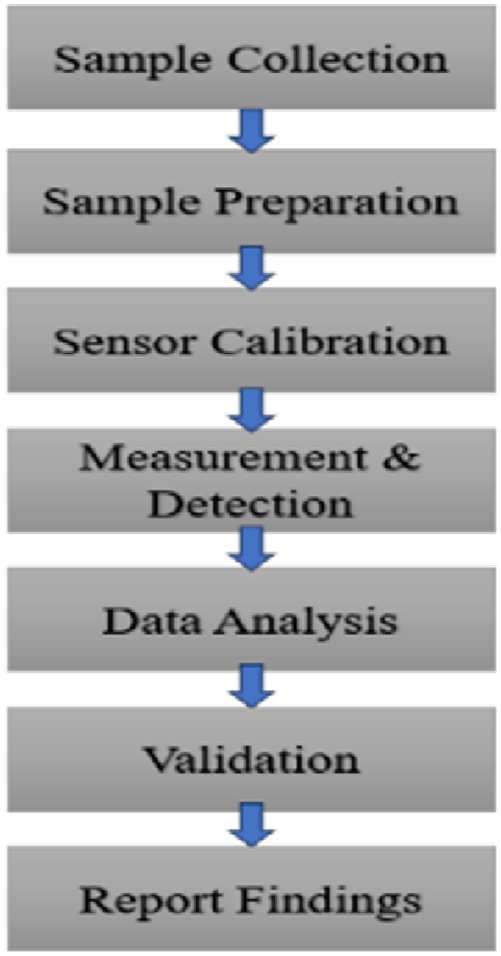
Detection steps for analyzing alcohol in commercial food samples.

To apply the heptagonal core PCF sensor for detecting alcohol in real commercial food samples, the process begins with collecting a variety of samples, such as alcoholic beverages or food with ethanol-based preservatives. After proper sample preparation, the sensor is calibrated using known concentrations of methanol and ethanol. The prepared samples are then analyzed with the sensor to detect and differentiate the alcohols based on their refractive index variations. The data gathered from the sensor is compared with conventional methods like gas chromatography to validate its accuracy and reliability in real-world applications shown in Fig. 9. Finally, the results are documented to highlight the sensor's effectiveness and potential for improving alcohol detection in commercial food products. Fig. 9 Illustrates the workflow for applying the PCF sensor in detecting alcohol in food products, from sample preparation to data analysis.

The limitations of THz PCF sensors in alcohol detection include challenges in precise fabrication, which can impact sensitivity and performance. Additionally, maintaining consistent sensor calibration in real-world conditions, such as varying temperatures and environmental factors, can be difficult. High costs of THz components and limited availability of commercial THz sources further restrict widespread adoption in practical applications. While this study demonstrates the sensor's high sensitivity and specificity in detecting methanol and ethanol under controlled conditions, future work could involve validating its performance with real commercial food samples, such as alcoholic beverages or foods preserved with ethanol-based ingredients. To ensure accurate readings in complex matrices, additional calibration may be necessary to account for potential interferences from food constituents, including sugars, proteins, and other additives. Experimental testing on real samples would provide valuable data on the sensor's robustness in practical applications, as well as insights into any sensitivity adjustments required. Key challenges include maintaining sensor calibration in variable environmental conditions and addressing the high costs and limited availability of commercial THz sources, which may affect widespread adoption. Incorporating such real-world data in future studies would further solidify the sensor's utility for alcohol detection in food safety applications.

## 5 Conclusion

The primary objective of this research is to develop a novel heptagonal core photonic crystal fiber (PCF) sensor capable of detecting both methanol and ethanol simultaneously with high sensitivity. Methanol is extremely harmful to living organisms, potentially causing serious illnesses such as cataracts, organ damage, or even death, while ethanol is generally safe for human consumption in moderation. Misidentifying methanol as ethanol can lead to severe toxicity and fatal consequences, making accurate detection vital for public safety. The proposed sensor, designed using COMSOL Multiphysics, Key metrics include a high relative sensitivity (RS) of 95.72 % for methanol and 97.55 % for ethanol, along with low effective material loss (EML) values of 0.0066 cm⁻^1^ and 0.0044 cm⁻^1^, respectively. Furthermore, the sensor features optimized Effective Areas (EA) of 3.54 × 10⁻⁸ m^2^ for methanol and 3.92 × 10⁻⁸ m^2^ for ethanol, and small spot sizes (SP) of 2.12 × 10⁻⁴ μm and 2.28 × 10⁻⁴ μm, respectively. This high level of precision and performance in the THz range ensures reliable detection, which is critical for improving safety in industrial and commercial environments. The ability to detect both methanol and ethanol simultaneously at such high sensitivity distinguishes this sensor from existing technologies, contributing to enhanced public health protection and industrial monitoring. The proposed heptagonal core PCF sensor offers significant real-world applications, particularly in industrial monitoring and public safety. Its high sensitivity and precision in detecting methanol and ethanol make it crucial for preventing methanol poisoning in industries that handle alcohol-based substances. Additionally, it enhances safety by ensuring accurate chemical identification in environments where the risk of contamination or accidental exposure is high. For future research, efforts should focus on optimizing the fabrication process to make the sensor commercially viable. Investigating materials with higher thermal and mechanical stability could enhance durability, while scaling up production methods would enable widespread industrial use. Additionally, exploring integration with real-time monitoring systems could further expand its practical applications.

## CRediT authorship contribution statement

**Most. Momtahina Bani:** Data curation, Investigation, Methodology, Software, Writing – original draft. **Khalid Sifulla Noor:** Data curation, Investigation, Methodology, Software, Writing – original draft. **A.H.M. Iftekharul Ferdous:** Conceptualization, Project administration, Resources, Supervision, Validation, Visualization, Writing – review & editing. **Md. Asaduzzaman Shobug:** Supervision, Validation, Visualization, Writing – review & editing.

## Declaration of competing interest

The authors declare that they have no known competing financial interests or personal relationships that could have appeared to influence the work reported in this paper.

## References

[1] Weir K. (1995). Optical fiber sensor technology. J. Mod. Opt..

[2] Hartog A., Gamble G. (1991). Photonic distributed sensing. Phys. World.

[3] Sharma M., Mishra S.K., Ung B. (2019, September). Ultra-sensitive and large dynamic range refractive index sensor utilizing annular core photonic crystal fiber. Photonic Fiber and Crystal Devices: Advances in Materials and Innovations in Device Applications XIII.

[4] Mishra S.K., Azad S., Mac-Thiong J.M., Ung B. (2020, September). Frontiers in Optics.

[5] Kirchhof J., Kobelke J., Schuster K., Bartelt H., Iliew R., Etrich C., Lederer F. (2006).

[6] Li H., Zhao C., Li J., Zheng C., Xu H., Xu W., Yao J. (2024). Broadband all-dielectric meta-lenses with terahertz full-Stokes polarization detection behavior. Opt Express.

[7] Liu H., Zhong H., Karpowicz N., Li X., Redo A., Chen Y., Jin Y.S. (2005, October). 2005 International Topical Meeting on Microwave Photonics.

[8] Al-Douseri F.M., Chen Y., Zhang X.C. (2006). THz wave sensing for petroleum industrial applications. Int. J. Infrared Millimet. Waves.

[9] Pawar A.Y., Sonawane D.D., Erande K.B., Derle D.V. (2013). Terahertz technology and its applications. Drug Invent. Today.

[10] Zhaoxin L., Dehua L., Science T. (2015). Identification of terahertz fingerprint spectra extracted from Gas‐Fat coal. Int. J. Terahertz Sci. Technol..

[11] Sultana J., Islam M.S., Ahmed K., Dinovitser A., Ng B.W.H., Abbott D. (2018). Terahertz detection of alcohol using a photonic crystal fiber sensor. Applied optics.

[12] Zhi-guo Z., Fang-di Z., Min Z., Pei-da Y. (2008). Gas sensing properties of index-guided PCF with air-core. Opt Laser. Technol..

[13] Møller U., Sørensen S.T., Larsen C., Moselund P.M., Jakobsen C., Johansen J., Bang O. (2012). Optimum PCF tapers for blue-enhanced supercontinuum sources. Opt. Fiber Technol..

[14] Yin G., Wang Y., Liao C., Sun B., Liu Y., Liu S., Zhong X. (2014). Simultaneous refractive index and temperature measurement with LPFG and liquid-filled PCF. IEEE Photon. Technol. Lett..

[15] Cai S., Yu S., Wang Y., Lan M., Gao L., Gu W. (2015). Hybrid dual-core photonic crystal fiber for spatial mode conversion. IEEE Photon. Technol. Lett..

[16] Naeem K., Chung Y., Kwon I.B. (2017). Highly sensitive two-dimensional bending vector sensor using an elliptic two-core PCF. IEEE Photon. Technol. Lett..

[17] Jing J.Y., Wang Q., Wang B.T. (2018). Refractive index sensing characteristics of carbon nanotube-deposited photonic crystal fiber SPR sensor. Opt. Fiber Technol..

[18] Aporta I., Quintela M.A., López-Higuera J.M. (2019). Switchable dual-wavelength mode-locked fiber laser source for in-PCF parametric frequency conversion applied to CARS microscopy. J. Lightwave Technol..

[19] Kotsina N., Belli F., Gao S.F., Wang Y.Y., Wang P., Travers J.C., Townsend D. (2019). Ultrafast molecular spectroscopy using a hollow-core photonic crystal fiber light source. The journal of physical chemistry letters.

[20] Danlard I., Akowuah E.K. (2020). Assaying with PCF-based SPR refractive index biosensors: from recent configurations to outstanding detection limits. Opt. Fiber Technol..

[21] Sen S., Abdullah-Al-Shafi M., Kabir M.A. (2020). Hexagonal photonic crystal Fiber (H-PCF) based optical sensor with high relative sensitivity and low confinement loss for terahertz (THz) regime. Sensing and Bio-Sensing Research.

[22] Islam M.R., Iftekher A.N.M., Hasan K.R., Nayen M.J., Islam S.B., Islam R., Tasnim Z. (2021). Surface plasmon resonance based highly sensitive gold coated PCF biosensor. Appl. Phys. A.

[23] Yin Z., Jing X., Bai G., Wang C., Liu C., Gao Z., Li K. (2022). A broadband SPR dual-channel sensor based on a PCF coated with sodium-silver for refractive index and temperature measurement. Results Phys..

[24] Iftekharul Ferdous A.H.M., Noor S.Z.E., Kalpana Devi P., Kavitha K.R., Anitha G., Jayakumar T., Ahammad S.H. (2024). Unlocking insights of oil derivatives with terahertz spectrum analysis: the hybrid refractive index rectangular core photonic crystal fiber perspective sensing. J. Opt..

[25] Noor K.S., Ferdous A.I., Bani M.M., Sadeque M.G. (2024). Floral-core terahertz photonic crystal fiber bio-sensor: an exclusive approach to recognizing milk from various animal species. Applied Food Research.

[26] Shobug M.A., Noor K.S., Sagaya Raj A.G., Ramkumar G., Padmanaban P., Mallan S., Rashed A.N.Z. (2024). Fuel quality assurance based on hybrid hexagonal circular hollow core PCF sensing through management of terahertz region operation. J. Opt..

[27] Kanmani R., Ahmed K., Roy S., Ahmed F., Paul B.K., Rajan M.M. (2019). The performance of hosting and core materials for slotted core Q-PCF in terahertz spectrum. Optik.

[28] Kundu D., Faruk M.O., Khandakar K., Ferdous A.I., Bani M.M., Noor K.S. (2024). Terahertz photonic crystal fiber sensor: mvaluating performance for chemical identification. Int. Commun. Heat Mass Tran..

[29] Vale A. (2007). Ethanol. Medicine.

[30] Tephly T.R. (1991). The toxicity of methanol. Life Sci..

[31] Dashtban Z., Ramtinfard S., Kakesh N., Saghaei H. (2024). Design and characterization of an ultra-sensitive photonic crystal fiber-based sensor for precise detection of alcohols in the THz regime. Results in Optics.

[32] Islam M.S., Sultana J., Ahmed K., Islam M.R., Dinovitser A., Ng B.W.H., Abbott D. (2017). A novel approach for spectroscopic chemical identification using photonic crystal fiber in the terahertz regime. IEEE Sensor. J..

[33] Habib M.A., Anower M.S., Abdulrazak L.F., Reza M.S. (2019). Hollow core photonic crystal fiber for chemical identification in terahertz regime. Opt. Fiber Technol..

[34] Rahaman M.E., Jibon R.H., Mondal H.S., Hossain M.B., Bulbul A.A.M., Saha R. (2021). Design and optimization of a PCF-based chemical sensor in THz regime. Sensing and Bio-Sensing Research.

[35] Nader A. Issa, Martijn A. van Eijkelenborg, Matthew Fellew, Felicity Cox, Geoff Henry, Maryanne C. J. Large (2004). Fabrication and study of microstructured optical fibers with elliptical holes. Opt Lett..

[36] Bostan C.G., de Ridder R.M., Gadgil V.J., Kuipers L., Driessen A. (2003). Proceddings Symposium IEEE/LEOS Benelux Chapter, 2003.

